# Supplementary material for: Optimising the tilt increment for in situ cryo-electron tomography
Source: eLife. 2026 Aug 24;15:RP111639. doi: 10.7554/eLife.111639 (PMC13502964; doi:10.7554/eLife.111639)
Supplement: Peer review history

# Editor report: eLife Assessment

**DOI:** 10.7554/eLife.111639.3.sa0

**Authors:** Axel T Brunger

**Affiliations:** Stanford University School of Medicine, Howard Hughes Medical Institute United States

**Keywords:** Convincing, Valuable

This **convincing** contribution addresses a question of practical importance: when collecting tilt-series data, what is the optimal angular step size between successive tilt images? The work provides **valuable** practical insights into cryo-ET data acquisition by demonstrating that balancing two competing demands—sufficient dose per individual tilt image and fine angular sampling—is essential to achieve high-quality tomographic reconstructions. They demonstrate that tilt-series acquired with finer increments (1-3°) yield superior alignment accuracy and improved template-matching performance.

# Referee report: Reviewer #1 (Public review)

**DOI:** 10.7554/eLife.111639.3.sa1

This work addresses a question of practical importance that had never been systematically analysed in the cryo-ET field: when collecting tilt-series data, what is the optimal angular step size between successive tilt images? Due to the upper limit in electron exposure (100 - 150 e⁻/Å²), this question is important, since finer angular sampling improves attainable reconstruction resolution (Crowther criterion) but reduces the signal-to-noise ratio of each individual image, potentially compromising both image quality and the ability to computationally align successive frames. To address this, the authors designed a thorough benchmarking study comparing five tilt increments (1°, 2°, 3°, 5°, and 10°) while keeping the total dose and tilt range constant. They evaluated the consequences at every stage of the cryo-ET workflow - from raw image quality and tilt-series alignment, through template matching for ribosome detection, to high-resolution subtomogram averaging - with the goal of providing the community with an evidence-based recommendation for data acquisition.

The manuscript is well written, and the experimental design is carefully thought out. The work provides valuable practical insights into cryo-ET data acquisition by demonstrating that balancing two competing demands - sufficient dose per individual tilt image and fine angular sampling - is essential to achieve high-quality tomographic reconstructions. The identification of a practical optimum at 3° tilt increment is the key contribution of the work. It will be interesting to see in the future whether this optimum shifts for smaller molecular targets, and how emerging tilt interpolation strategies such as cryoTIGER may interact with the choice of experimental angular increment.

Comments on revised version.

Well done! I really like the manuscript and from my point of view it's an excellent piece of work and super useful for the community. Thank you so much for the meticulous work!

# Referee report: Reviewer #2 (Public review)

**DOI:** 10.7554/eLife.111639.3.sa2

The determination of macromolecular structures directly within their native cellular environment is becoming increasingly routine, making standardized data collection strategies essential. In this manuscript, Tuijtel et al. provide a timely and valuable contribution by benchmarking key acquisition parameters and establishing practical guidelines for in situ cryo-electron tomography (cryo-ET). Critically, the authors present a systematic framework for optimizing data collection to achieve the highest attainable resolution.

Using Dictyostelium cells as a model system, the authors generate multiple datasets at a constant total dose while varying the tilt increment. They demonstrate that tilt-series acquired with finer increments (1-3°) yield superior alignment accuracy and improved template-matching performance, resulting in higher-quality reconstructions than those collected with coarser increments (5° or above). Furthermore, the authors show that for subtomogram averaging, a 3° tilt increment outperforms all other conditions tested, particularly after per-particle refinement as implemented in M.

Comments on revised version.

The authors have addressed all my concerns, and I have no further issues.

# Author comment: Author response

**DOI:** 10.7554/eLife.111639.3.sa3

**Authors:** Maarten Willem Tuijtel, Tomáš Majtner, Beata Turoňová, Martin Beck

**Affiliations:** Max Planck Institute of Biophysics Frankfurt Germany; Max Planck Institute of Biophysics Frankfurt Germany; Max Planck Institute of Biophysics Frankfurt Germany; Max Planck Institute of Biophysics Frankfurt Germany

The following is the authors’ response to the original reviews.

**Public Reviews:**

**Reviewer #1 (Public review):**

This work addresses a question of practical importance that had never been systematically analysed in the cryo-ET field: when collecting tilt-series data, what is the optimal angular step size between successive tilt images? Due to the upper limit in electron exposure (100 - 150 e^-^/Å^2^), this question is important, since finer angular sampling improves attainable reconstruction resolution (Crowther criterion) but reduces the signal-to-noise ratio of each individual image, potentially compromising both image quality and the ability to computationally align successive frames. To address this, the authors designed a thorough benchmarking study comparing five tilt increments (1°, 2°, 3°, 5°, and 10°) while keeping the total dose and tilt range constant. They evaluated the consequences at every stage of the cryo-ET workflow - from raw image quality and tilt-series alignment, through template matching for ribosome detection, to high-resolution subtomogram averaging - with the goal of providing the community with an evidence-based recommendation for data acquisition.

The manuscript is well written, and the experimental design is carefully thought out. The work provides valuable practical insights into cryo-ET data acquisition by demonstrating that balancing two competing demands - sufficient dose per individual tilt image and fine angular sampling - is essential to achieve high-quality tomographic reconstructions. The identification of a practical optimum at 3° tilt increment is the key contribution of the work. It will be interesting to see in the future whether this optimum shifts for smaller molecular targets, and how emerging tilt interpolation strategies such as cryoTIGER may interact with the choice of experimental angular increment.

The conclusions of this paper are mostly well supported by data, but some aspects of data analysis need to be clarified and/or extended, including:

(1) Line 109: The authors state that the tilt range was kept at ± 60° relative to the lamella plane. Assuming a typical lamella pre-tilt of ~10°, the absolute stage tilt would approach its mechanical limit. Two clarifications would be appreciated: (a) What was the average pre-tilt across all lamellae? (b) How many dark tilt images, if any, were excluded during tomogram reconstruction?

We thank the reviewer for asking for further clarification. For all our datasets, the pre-tilt of the stage was +8° with the lamella untilted under the e-beam, resulting in a tilt range of -52° to + 68°, thereby not reaching the mechanical limit, which is 70° for our microscope stage.

Regarding “dark tilt images”, for most datasets, we did not need to remove many tilt images. However, we now noticed notably more absence of images from higher tilt values for the 1° dataset (see SFig 1). When analysing further, we noticed that for this dataset, we did not actively remove many images prior to tomogram reconstruction, but rather that they were not acquired in the first place by SerialEM. During acquisition, SerialEM performs various safeguarding checks that can abort the acquisition of a tilt series (or of a single branch). As this seems predominantly a problem for the 1° tilt-increment dataset, we have decided to add this to the manuscript as follows, including the figure as new SFig 1.

In the main text:

“For most datasets, image acquisition was largely complete, with the exception of the 1° dataset, which showed a markedly higher proportion of missing images at high tilt angles (SFig. 1). Closer inspection revealed that many of these images were not acquired, as SerialEM applies built-in safeguards (e.g. autofocus inconsistency or insufficient image counts) that can abort a tilt-series branch before completion.”

(2) Line 148: "When analysing tomographic volumes, we found that tomograms from data with a smaller increment displayed higher SNR values (see Fig. 2B)." It would be helpful to specify which comparisons are statistically meaningful (e.g. Mann-Whitney U test?). While the difference between 1° and 2° appears pronounced, the differences between 2°, 3°, and 5° seem minimal. From my point of view, reporting the mean SNR values +/- standard deviations for each condition would already indicate some significance. Furthermore, since SNR is expected to depend on lamella thickness, it should be clarified whether the average lamella thickness is comparable across the five datasets.

We have now calculated the mean and standard deviation of the tomogram SNR, as follows:

**Author response table 1.** 

1°	mean=0.0612	std=0.0259	n=63
2°	mean=0.0406	std=0.0091	n=119
3°	mean=0.0381	std=0.0135	n=119
5°	mean=0.0322	std=0.0117	n=122
10°	mean=0.0145	std=0.0055	n=89

Furthermore, we performed a statistical significance test. Kruskal-Wallis test confirmed significant differences in SNR across tilt increments (H=270.97, p<0.001). Pairwise Mann-Whitney U tests with Bonferroni correction revealed significant differences between all pairs except 2° and 3° (p=0.093), suggesting these two conditions indeed yield comparable SNR.

Lastly, we have now added data regarding local lamella thickness for all tilt-series used in the study, as displayed in SFig. 4.

We incorporated this in the manuscript as follows.

In the main text:

“When analysing tomographic volumes, we found that tomograms from data with a smaller increment displayed higher SNR values (see Fig. 2B and Supplementary Note), whilst showing a similar lamella thickness distribution (see SFig. 4A).”

and:

“Firstly, we selected ca. 20 tomograms per condition, based on tomogram content and local lamella thickness [31] (for more details, see Methods and SFig. 4B).”

As a supplementary note:

“As the tomogram SNR distribution of particularly the 2° and 3° dataset showed similar SNR distributions, we performed formal significance testing for the data in this panel (see Fig. 2B). Kruskal-Wallis test confirmed significant differences across conditions (H=270.97, p<0.001); pairwise Mann-Whitney U tests with Bonferroni correction revealed all pairs were significantly different except 2° vs. 3° (p=0.093), indicating comparable SNR for these two tilt increments.”

And, adding the test in the Methods:

“To quantify differences in signal-to-noise ratio (SNR) across tilt increment conditions, a non-parametric Kruskal-Wallis test was performed as an omnibus test of the null hypothesis that all groups are drawn from the same distribution. Because SNR distributions were not assumed to be normal, and sample sizes differed across conditions, non-parametric tests were used throughout. Following the omnibus test, all 10 pairwise comparisons between conditions were assessed using two-sided Mann-Whitney U tests. To control for multiple comparisons, raw p-values were adjusted using the Bonferroni correction (multiplied by the number of comparisons, n=10, capped at 1.0). Statistical significance was defined as a Bonferroni-corrected p-value below 0.05. All analyses were performed in Python using the scipy.stats module.”

(3) Line 167: "Indeed, the variation in maximum resolution correlates with lamella thickness across all datasets (see Fig. 2F)." The reported R^2^ values of 0.30 (1°), 0.38 (2°), 0.66 (3°), 0.61 (5°), and 0.60 (10°) reveal a notably weak linear relationship for the finer tilt increments. It is also difficult to assess whether the lamella thickness distributions are comparable across conditions from the current figures - visually, the 1° dataset appears to be based on thinner lamellae, while the 10° dataset appears to include thicker samples. A histogram of lamella thickness distributions for each condition, provided as supplementary material, would greatly aid interpretation. Given this thickness dependency, reporting mean +/- standard deviation of lamella thickness per condition is highly appreciated.

We have added the full lamella thickness distribution per dataset now in SFig. 4.

The apparent weaker relationship between resolution fit and local lamella thickness for the 1 dataset seems to be largely apparent to the few very thin data points in this data (for more clarity, see the same data plotted separately in Author response image 1). We speculate that this is due to even less signal in these very thin and very low-dose images.

**Author response image 1.**

(4) Figure 4: It should be specified which tomogram subsets were used for the Rosenthal-Henderson analysis, whether lamella thickness was taken into account in the subset selection, and whether ribosomes too close to the lamella edges were excluded. Finally, linear fits should be displayed across the full x-axis range for all tilt increments to facilitate direct visual comparison.

We have described the process of tomogram subset selection in detail in the Methods section Template matching and 3D classification. To further add clarity, we have incorporated the local lamella distribution plots for the full data, as well as specifically for the tomograms subjected to TM and STA in SFig. 4.

Regarding the linear fits, we respectfully disagree with this suggestion. Displaying the linear fits only over the range used for their calculation avoids implying that the linear relationship extends beyond the measured data, and in our view produces a clearer figure.

(5) General: Were ribosomes located at the lamella edges excluded from the analysis? As demonstrated in the authors' own prior work (Tuijtel et al., Science Advances, 2024), Ga-FIB milling induces structural damage at the lamella surfaces. To exclude the influence on the STA results, particles near the lamella edges should be removed prior to analysis, and the criteria for this exclusion should be stated explicitly.

We have not excluded any ribosomes from close to the surface. As we still treated all data the same for each condition, we anticipate that the results of the comparison reported here still hold true.

The aim of the authors was to provide the cryo-ET community with an evidence-based recommendation for the choice of tilt increment, and they largely succeeded in this goal. The identification of 3° as a practical optimum - balancing sufficient dose per tilt image for effective per-particle refinement with fine enough angular sampling for accurate tilt-series alignment - is well supported by the data and consistent across the multiple quality metrics employed. The conclusion that coarser increments (5° and 10°) compromise tomogram quality, template matching accuracy, and STA resolution is robust and clearly demonstrated. However, the conclusion rests entirely on a single biological system using ribosomes as the sole molecular target, which are exceptionally favourable due to their abundance, size, and electron contrast. Whether the identified optimum holds for smaller, lower-abundance, or lower-contrast targets remains an open question.

In future, it would be particularly interesting to test whether emerging tilt interpolation strategies, such as cryoTIGER, which is particularly intriguing, can effectively compensate for coarser experimental angular sampling in post-processing. Here, the optimal experimental increment may shift, and the interaction between these two approaches represents a promising direction for future work. More broadly, as cryo-ET datasets grow larger and public repositories expand, the practical tradeoffs between acquisition time, data storage, and structural quality identified here will become increasingly relevant to the field.

We agree with the reviewer and thank them for this positive assessment. An interesting note to the use of cryoTIGER in particular is that it uses already aligned tilt-series as an input, and it therefore is unlikely to overcome severe alignment issues associated with large tilt-increments.

**Reviewer #2 (Public review):**

The determination of macromolecular structures directly within their native cellular environment is becoming increasingly routine, making standardized data collection strategies essential. In this manuscript, Tuijtel et al. provide a timely and valuable contribution by benchmarking key acquisition parameters and establishing practical guidelines for in situ cryo-electron tomography (cryo-ET). Critically, the authors present a systematic framework for optimizing data collection to achieve the highest attainable resolution.

Using Dictyostelium cells as a model system, the authors generate multiple datasets at a constant total dose while varying the tilt increment. They demonstrate that tilt-series acquired with finer increments (1-3°) yield superior alignment accuracy and improved template-matching performance, resulting in higher-quality reconstructions than those collected with coarser increments (5° or above). Furthermore, the authors show that for subtomogram averaging, a 3° tilt increment outperforms all other conditions tested, particularly after per-particle refinement as implemented in M.

Overall, the manuscript is clearly written, and the conclusions are well supported by the data presented. I have no major concerns. There are some minor points that the authors should address, including:

(1) The phrase "electron optical density distribution" (line 31, Introduction) should be revised to "electrostatic potential" or "Coulomb potential distribution," which more accurately reflects what is measured in cryo-EM/ET.

We thank the reviewer for this correction and have adjusted it in the text:

“It captures the 3-dimensional (3D) electrostatic potential of the specimen under scrutiny and enables the structural analysis of macromolecular complexes within their native context.”

(2) The authors state that the maximum tolerable electron dose is approximately 100-150 e^-^/Å^2^ (line 34, Introduction). This is an oversimplification, as bacterial specimens, for example, have been shown to tolerate doses of 200 e^-^/Å^2^ or higher (see Breigel et al., PNAS, 2009; https://www.pnas.org/doi/10.1073/pnas.0905181106#T1). The statement should be revised to reflect this variability.

We adjusted this statement to now read:

“One of these is the maximum electron dose that can be applied to biological specimens before irreversible damage occurs, which is about 100-150 e-/Å2 for most eukaryotic cells.”

(3) Lines 56-57: The authors do not cite their own prior work benchmarking tilt-series acquisition strategies on in vitro samples. This earlier study provides important context and should be referenced and briefly discussed.

We assume the reviewer meant this study: Turonova et al., Nat. Comms. (2020). We have now added and discussed this reference as follows:

“Since accumulated radiation dose progressively degrades high-resolution information, this motivated the development of the dose-symmetric tilt scheme, which prioritizes acquisition of low-tilt images early to better preserve high-resolution information [11, 16].”

**Recommendations for the authors:**

**Reviewer #1 (Recommendations for the authors):**

(1) Line 159: "Surprisingly though, the resolution to which the CTF was fitted was similar for all conditions, despite an 8-fold increase in dose (see Fig. 2B, E)." The reference to Figure 2B at this point is unclear.

We thank the reviewer for pointing this out, we have removed the reference to panel B.

(2) Line 162: "As the data shown in Fig. 2D-F pertains to images of the untilted specimen, ..." For clarity, this should also be stated explicitly in the figure caption.

We have added this to the figure legend; “both estimated with Gctf on projection images of the sample at effective zero-tilt position.”

(3) Figure 3: These are compelling results, and the 3D classification outcomes provide an excellent visual representation of the quantitative data shown in Figure 3. Including (some or all) of the initial five classes in the figure would further strengthen this already convincing presentation. Additionally, applying a uniform extraction threshold (e.g., z-score of 3.75 or 5) across all tilt increments would facilitate a more direct comparison. But this is really a minor remark, the authors and the editors may judge if the current presentation is already sufficient.

We have adjusted Figure 3 according to the reviewer’s recommendation:

We referenced this in the main text as:

“To ensure similar data processing strategies for all conditions, extraction thresholds were also lowered for the 1, 2 and 3° conditions, and 3D classification was performed to filter out the junk particles (see Fig. 3 A and SFig. 8). “

In order to directly compare the particle extraction, we have already carried out such a uniform extraction threshold, with a z-score threshold of 5 (apart for the 10° data, where this was not possible). This extraction was then used for the 3D classification that led to the TM analysis and further STA investigations.

**Reviewer #2 (Recommendations for the authors):**

(1) Supplementary Figure 1: To improve accessibility for a broader readership, the authors should annotate or highlight the key organelles and protein complexes visible in the tomographic slices.

We thank the reviewer for this suggestion, but adding arrowheads made this figure too crowded in our opinion. Furthermore, for most of the panels, the mentioned features of interest is centred in the image panel, which should make identification straightforward.

(2) 'In situ' should be in italics throughout the text.

We have changed this.
